# Distribution of Causative Organisms in Infective Endocarditis and Their Associated Mortality Risk: A Single-Centre Study From North Wales, United Kingdom

**DOI:** 10.7759/cureus.90759

**Published:** 2025-08-22

**Authors:** Daniel E Ekwueme, Stephanie Dahr, Lauren Grinstead, Irfan Ali Rind

**Affiliations:** 1 Internal Medicine, Wrexham Maelor Hospital, Wrexham, GBR; 2 Cardiology, Wrexham Maelor Hospital, Wrexham, GBR

**Keywords:** blood cultures, coagulase-negative staphylococci, culture-negative infective endocarditis, epidemiology and biostatistics, infective endocarditis, mortality risk, north wales, polymicrobial infection, staphylococcus aureus endocarditis, viridans streptococci

## Abstract

Background

Infective endocarditis (IE) is a life-threatening infection of the endocardial surface, most commonly affecting native or prosthetic valves and intracardiac devices. Despite advancements in diagnosis and treatment, IE continues to carry a high mortality risk. Blood cultures remain a cornerstone of diagnosis, typically yielding positive results and enabling identification of the causative organisms. *Staphylococcus aureus* is now recognised as the most prevalent pathogen, particularly in healthcare-associated and intravenous drug use-related cases, and is associated with worse outcomes. Other commonly implicated organisms include streptococci and enterococci. This study aimed to investigate the distribution of causative pathogens and their associated mortality risk in patients diagnosed with IE at the Wrexham Maelor Hospital, North Wales, United Kingdom.

Method

A retrospective cohort study was conducted using patient data from the Welsh Clinical Portal at the Wrexham Maelor Hospital, covering the period from June 2022 to May 2025. Patients were eligible for inclusion if they had a confirmed diagnosis of IE based on the modified Duke criteria. Statistical analysis was performed using Chi-squared or Fisher’s exact test to evaluate associations between pathogen type and mortality.

Results

A total of 50 patients with positive blood cultures were included. *Staphylococcus aureus* was the most frequently isolated organism (n=19; 38%), followed by polymicrobial infections (n=7; 14%), *Staphylococcus epidermidis* (n=4; 8%), and *Enterococcus faecalis* (n=3; 6%). *Streptococcus sanguinis*, *Escherichia coli*, and *Haemophilus parainfluenzae* each accounted for 4% (n=2), with the remaining 2% (n=1) comprising less common pathogens. Twenty-three deaths were recorded, with the highest mortality observed in the culture-negative group (n=6). No statistically significant correlation was found between the pathogen type and mortality.

Conclusion

While *Staphylococcus aureus* was the most frequently isolated pathogen, the highest mortality occurred in patients with culture-negative IE. This may reflect the diagnostic and therapeutic challenges posed by the absence of microbiological confirmation, potentially delaying appropriate, targeted antimicrobial therapy. These findings highlight the importance of early diagnosis, empiric antimicrobial coverage, and timely treatment adjustments to improve outcomes in IE, particularly in culture-negative presentations.

## Introduction

Infective endocarditis (IE) is a serious and potentially fatal infection involving the endothelial lining of the heart, most often affecting native or prosthetic heart valves [1-4]. Although relatively uncommon, IE presents significant clinical challenges due to its complex pathophysiology, diagnostic ambiguity, and high morbidity and mortality rates [2,5,6,7]. The global burden of IE appears to be rising, with incidence estimates ranging from three to 10 cases per 100,000 of the population annually [3,6,7]. This increase is largely attributed to demographic and clinical shifts, including the ageing population, increased use of prosthetic valves and intracardiac devices, and the growing prevalence of healthcare-associated infections and invasive medical interventions [2,3,4,7].

Timely identification of the causative organism remains central to the diagnosis and management of IE. Patients with IE will typically, but not always, have positive blood cultures [8-10]. As such, blood cultures serve as the primary tool for identifying pathogens and guiding antimicrobial therapy. However, culture-negative endocarditis persists as a diagnostic hurdle, often resulting from prior antibiotic use or fastidious organisms, and is associated with delays in appropriate therapy and worse outcomes [8,9,11].

*Staphylococcus aureus *has become the predominant causative agent in both hospital-acquired and intravenous drug use-associated IE [12-15]. This pathogen is linked with a particularly aggressive clinical course, including rapid valve destruction, systemic embolisation, and elevated mortality risk [13,14,16,17]. Other frequently implicated organisms include viridans group streptococci, enterococci, and coagulase-negative staphylococci, each carrying distinct prognostic implications [12,14,18]. Despite improvements in imaging modalities and therapeutic strategies, mortality rates for IE remain substantial, often exceeding 20% in contemporary cohorts [3,15,19,20].

The management of IE primarily involves prolonged intravenous antimicrobial therapy, guided by the identified pathogen and its antimicrobial susceptibility profile [18,20,21]. Empiric broad-spectrum antibiotics are typically initiated following blood culture collection, with subsequent de-escalation and refinement once microbiological confirmation is obtained [11,17]. Treatment approaches vary significantly depending on whether the infection involves a native or prosthetic valve. Prosthetic valve endocarditis often necessitates extended treatment durations and combination antibiotic regimens due to the increased risk of antimicrobial resistance and biofilm formation [20,22]. In selected patients, early surgical intervention may be warranted, particularly in cases complicated by heart failure, persistent or uncontrolled infection, systemic embolization, or large vegetations [16,19]. Prompt surgical evaluation is especially critical in prosthetic valve IE or when infections are caused by highly virulent organisms such as *S. aureus* or fungal pathogens [12,10,23].

Understanding the pathogen-specific risks and outcome patterns in IE is critical for guiding empirical treatment decisions, risk stratification, and public health interventions. However, existing data are often limited by geographic variability and small sample sizes [1,2,11,16].

This retrospective study aimed to characterise the microbiological spectrum of IE in the Wrexham Maelor Hospital and examine the relationship between individual pathogen profiles and patient mortality outcomes. 

## Materials and methods

Study design and setting

A retrospective cohort study was conducted at the Wrexham Maelor Hospital, North Wales, UK, examining cases of IE diagnosed between June 2022 and May 2025. Patient data were extracted from the Welsh Clinical Portal, including demographics, blood culture results, and in-hospital mortality outcomes. Echocardiographic findings were obtained through the Centricity Cardiac Workflow system, encompassing both transthoracic and transoesophageal echocardiography reports.

Inclusion and exclusion criteria

Adult patients (≥18 years) with clinically suspected or confirmed IE, as defined by the modified Duke's criteria, the presence of positive blood cultures, and/or echocardiographic evidence consistent with IE, were included in this study. Patients with incomplete data and without echocardiographic data were excluded from the pathogen-specific analysis. 

Statistical analysis

The distribution of causative pathogens and associated mortality rates were tabulated. Statistical analyses were performed using IBM SPSS Statistics for Windows, Version 31 (Released 2025; IBM Corp., Armonk, New York, United States). The association between the identified pathogens and mortality outcomes was assessed using Pearson’s Chi-squared test or Fisher’s exact test, depending on expected frequencies. A p-value <0.05 was considered statistically significant.

Descriptive statistics were used to summarise demographic and microbiological data. Bar charts and heat maps were employed to visualise pathogen prevalence and mortality patterns, enhancing the interpretation of organism-specific outcome disparities.

## Results

As shown in Table 1, out of a total of 84 patients, 59.5% (n=50) had confirmed positive blood cultures, while 34.5% (n=29) were classified as culture-negative IE. The remaining 6% (n=5) did not undergo blood culture testing during their admission.

**Table 1:** Summary of pathogen-specific clinical outcomes, associated mortality rates, and results of statistical analyses (n=84)

Pathogen	Total cases (n)	Percentage of cases (%)	Deaths (n)	Mortality rate (%)	p-value
Streptococcus sanguinis	2	2.4	2	100	0.066
Polymicrobial	7	8.3	3	42.9	0.073
Staphylococcus aureus	19	22.6	4	21	0.989
Streptococcus gallolyticus	1	1.2	1	100	0.262
Streptococcus dysgalactiae	1	1.2	1	100	0.262
Granulicatella elegans	1	1.2	1	100	0.262
Staphylococcus epidermidis	4	4.8	2	50	1
Culture-negative	29	34.5	6	20.7	0.405
Enterococcus faecalis	3	3.6	1	33.3	1
Escherichia coli	2	2.4	1	50	0.458
Streptococcus pyogenes (Grp A)	1	1.2	1	100	1
Candida glabrata	1	1.2	0	0	1
Streptococcus infantarius	1	1.2	0	0	1
Fusobacterium necrophorum	1	1.2	0	0	1
Veillonella parvula	1	1.2	0	0	1
Streptococcus group G (STRG)	1	1.2	0	0	1
Haemophilus parainfluenzae (HPAF)	2	2.4	0	0	1
Streptococcus agalactiae group B (SAGA)	1	1.2	0	0	1
Streptococcus pneumoniae	1	1.2	0	0	1
No blood cultures done	5	6.0	0	0	0.319

Pathogen distribution

*S. aureus *was the most frequently isolated pathogen, identified in 22.6% (n=19) of the total cohort. Polymicrobial infections were the second most common, observed in 8.3% (n=7) of patients. Other organisms included *S. epidermidis* (n=4; 4.8%) and *Enterococcus faecalis* (n=3; 3.6%).

Several organisms, including *Streptococcus sanguinis*, *Escherichia coli*, and *Haemophilus parainfluenzae *were each isolated in 2.4% (n=2) of patients. A range of less commonly encountered pathogens were identified in single cases, such as *Streptococcus gallolyticus*, *Streptococcus pyogenes*, *Granulicatella elegans*, *Fusobacterium necrophorum*, *Veillonella parvula*, and *Candida glabrata*.

Mortality

**Figure 1:**
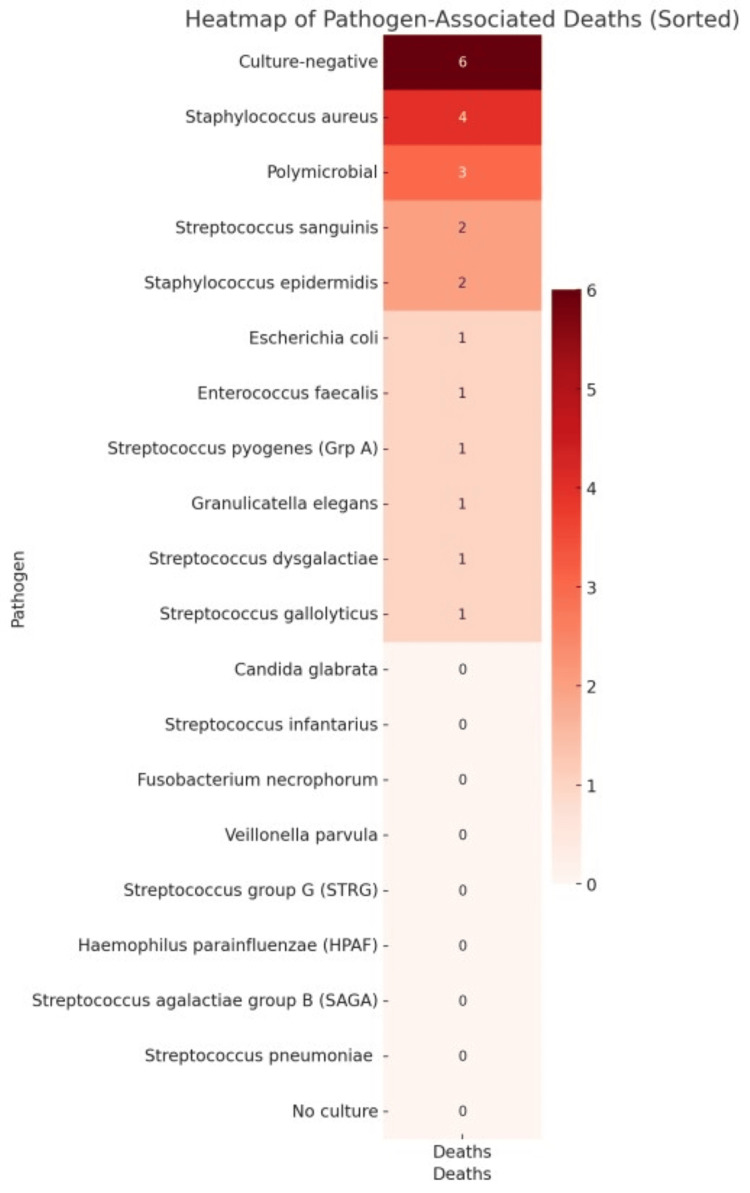
Heat map showing pathogen-associated mortality

**Figure 2:**
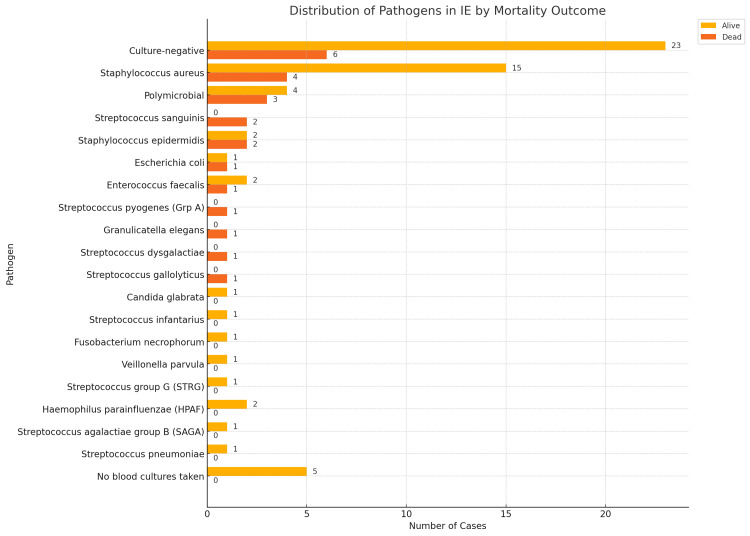
Distribution of pathogens in infective endocarditis (IE) by mortality outcome

As illustrated in Figures 1, 2, the highest number of deaths occurred in the culture-negative group (n=6), followed by *S. aureus* (n=4).

Overall mortality was 27.4%, indicating that 23 of 84 patients died during the study period. Mortality varied by pathogen. *S. aureus* was associated with a mortality of four out of 19 cases (21%), while polymicrobial infections and *Staphylococcus epidermidis* showed higher rates of mortality 42.9% (three out of seven cases) and 50% (two out of four cases), respectively. *Enterococcus faecalis *and *Escherichia coli *each had a mortality rate of 33.3% (one out of three cases) and 50% (one of out two cases), respectively. *Streptococcus sanguinis *was linked to mortality rate of 100% (two out of two cases). Culture-negative endocarditis had a mortality rate of 20.7% (six out of 29 cases), while no deaths occurred among patients without blood cultures.

Several pathogens, including *Candida glabrata*, *Fusobacterium necrophorum*, *Veillonella parvula*, *Haemophilus parainfluenzae*, *Streptococcus infantarius*, *Streptococcus pneumoniae*, and *Streptococcus* ​​*agalactiae*, were not associated with mortality. No statistically significant association was found between specific pathogens and mortality. 

## Discussion

This project presents a comprehensive evaluation of IE cases at the Wrexham Maelor Hospital over three years, focusing on the distribution of causative pathogens and their associated mortality, determined using post-discharge hospital records. Our dataset reinforces established trends while revealing organism-specific disparities in mortality that merit closer clinical scrutiny [2,6]. These findings underscore the importance of early identification, appropriate antimicrobial coverage, and targeted investigation, particularly in culture-negative and polymicrobial cases [8,10,11,13]. Although these trends were notable, the dataset did not demonstrate any statistically significant correlation between individual pathogens and mortality [6,7].

Clinical implications and treatment considerations

These findings highlight several important considerations for the management of IE at the Wrexham Maelor Hospital and similar institutions.

S. aureus

As the most common pathogen identified, *S. aureus* accounted for mortality in four out of 19 cases (21%) and is known for high complication rates, including embolisation and abscess formation. Early echocardiography is recommended in all cases of *S. aureus* bacteraemia, as up to 25% may involve an endocardial infection [4,5,14,20]. *S. aureus* remains a key concern in IE.

Streptococcus sanguinis

This cohort demonstrated that *Streptococcus sanguinis*, a viridans group streptococcus, was associated with 100% mortality (two out of two cases). Typically considered less aggressive, viridans streptococcal IE can lead to severe outcomes, particularly in high-risk patients. Early involvement of cardiology and prompt intervention are essential in suspected cases [15,18].

Polymicrobial endocarditis

With a mortality rate of 43% (three out of seven cases), polymicrobial IE was associated with particularly poor outcomes. Often linked to injection drug use or complex healthcare-associated infections, management requires broad-spectrum empiric antibiotics and frequently surgical intervention [23].

Coagulase-negative staphylococci (CoNS)

*S. epidermidis*, typically associated with prosthetic valve endocarditis (PVE), showed high mortality. PVE is linked to worse outcomes than native valve IE, often due to methicillin resistance and biofilm production, which frequently necessitate surgical management [14,24].

Enterococcus faecalis

Responsible for mortality in 33.3% of cases (one out of three cases), enterococcal IE affects older, comorbid patients and often requires synergistic antibiotic therapy due to intrinsic resistance patterns [14,15].

Fungal Endocarditis

A single case of *Candida glabrata* was documented with survival, contrary to the typical in-hospital mortality of over 50% seen in fungal IE. Management usually requires valve replacement and prolonged antifungal therapy, underscoring the need for high suspicion in immunocompromised or culture-negative patients [21].

Culture-Negative Endocarditis (BCNE)

Culture-negative cases constituted 34.5% (n=29) of the cohort, consistent with previous literature [10,11]. These often result from prior antibiotic exposure or fastidious organisms such as *Coxiella* or *Bartonella*. The elevated mortality may reflect diagnostic challenges related to the absence of microbiological confirmation, potentially leading to delays in initiating appropriate, targeted therapy and an increased risk of adverse outcomes. The high incidence of BCNE highlights the need for improved diagnostic strategies, prompt initiation of empiric and targeted antimicrobial therapy. Clinician education should emphasise obtaining blood cultures prior to antibiotic initiation in stable patients. Laboratory improvements, such as enhanced culture media, serological testing, and molecular diagnostics may increase the diagnostic yield in BCNE and should be incorporated into standard practice [8,11,13].

No-culture Cases

Five patients had no blood cultures taken, yet none died. These may reflect empiric treatment or delayed diagnosis. Nonetheless, obtaining blood cultures prior to initiating antibiotics in suspected IE remains a critical standard of care and should be reinforced through clinical policy [12,14].

Cases of polymicrobial IE and those caused by* S. aureus* are often linked to intravenous drug use, underscoring the importance of integrated harm reduction services. Programmes offering clean needle exchange, safer injection education, and addiction treatment have been shown to reduce IE incidence. Unsafe injection practices, including the use of saliva or non-sterile water, have been implicated in polymicrobial IE [23]. Integrating substance use treatment into IE care can improve outcomes and reduce reinfection risk [25].

Evidence increasingly supports the role of dedicated IE teams comprising infectious disease specialists, cardiologists, and cardiac surgeons. Hospitals should consider establishing formal multidisciplinary team (MDT) pathways, especially for high-risk pathogens. For example, automated alerts for *S. aureus* bacteraemia could trigger early imaging and surgical review. National policy could facilitate the development of endocarditis centres of excellence to manage complex cases requiring specialist care [12,14,18].

In summary, outcomes in IE vary considerably by pathogen, emphasising the need for prompt, individualised management. *S. aureus* remains the most prevalent and clinically severe organism, with high mortality and complication rates. *Streptococcus sanguinis*, though typically regarded as less virulent, was associated with unexpectedly poor outcomes, highlighting the potential severity of viridans streptococcal infections in vulnerable patients. Polymicrobial and fungal IE demonstrated particularly high mortality, often necessitating aggressive medical and surgical intervention. Coagulase-negative staphylococci and *Enterococcus faecalis* presented additional challenges due to potential for antimicrobial resistance. Culture-negative cases further complicate diagnosis and treatment, often delaying appropriate therapy. These findings underscore the critical importance of early, pathogen-specific risk stratification and multidisciplinary involvement including infectious diseases, cardiology, and cardiothoracic surgery. Importantly, even patients with traditionally less aggressive organisms require close monitoring, as the data set demonstrates no margin for complacency.

Limitations

As a single-centre retrospective study drawing on routine clinical documentation, our findings could be limited by possible missing data and miscoding.

The primary limitations of this study revolve around insufficient targeted research and the need for larger studies. The unexpectedly high mortality associated with *Streptococcus sanguinis* warrants further investigation. It remains unclear whether this was due to delayed diagnosis, comorbidities, or complications such as embolic events. However, the small number of cases limits definitive conclusions. A detailed case review or inclusion in larger multicentre studies may clarify whether viridans streptococci are becoming more virulent or if these outcomes were anomalous. Similarly, deeper analysis of polymicrobial cases to explore associations with intravenous drug use and identify organism combinations could inform tailored interventions for this subgroup.

Collaboration with other centres in Wales or contribution to national registries would provide a more representative picture. For example, confirming whether CoNS have consistently poorer outcomes, or whether *S. aureus* infections show similarly favourable results elsewhere, requires larger samples. Such data could inform national audits and support resource allocation, particularly given the high surgical burden associated with certain pathogens [14,24].

Future directions

Future research in IE should focus on three key areas to improve patient outcomes. Firstly, cases of intravenous drug use-related IE infections are on the rise and are strongly associated with *S. aureus* and polymicrobial infections. This particular subgroup requires deeper investigations to guide effective harm reduction initiatives. Secondly, a comprehensive examination of the risk factors and clinical consequences of polymicrobial endocarditis is essential, as this condition is linked to increased mortality and frequently associated with healthcare-related exposures and unsafe injection practices. Thirdly, evaluating the impact of multidisciplinary endocarditis teams on diagnosis, treatment, and surgical outcomes could underpin the development of consistent, evidence-based care pathways for high-risk groups. Strengthening research in these areas will not only enhance clinical decision-making but also support public health strategies aimed at reducing the burden of this complex and often fatal condition.

## Conclusions

This study offered a focused analysis of the microbiological profile and mortality patterns of IE within a regional centre in North Wales, and identified no statistically significant association between individual pathogens and mortality. Culture-negative endocarditis demonstrated the highest mortality, highlighting the clinical difficulty posed by the lack of microbiological confirmation. Delayed initiation of appropriate therapy may contribute to adverse outcomes in such cases. While statistical significance was not achieved, the findings reinforce the importance of early diagnosis and targeted antimicrobial treatment. Timely recognition, thorough microbiological investigation, and prompt intervention remain central to the effective management of IE, particularly given the increasing prevalence of culture-negative cases.
